# Characterization of a novel transplantable orthotopic rat bladder transitional cell tumour model

**DOI:** 10.1038/sj.bjc.6690741

**Published:** 1999-10

**Authors:** Z Xiao, T J McCallum, K M Brown, G G Miller, S B Halls, I Parney, R B Moore

**Affiliations:** Department of Experimental Surgery and Division of Urology, University of Alberta and Department of Surgery, Cross Cancer Institute, Edmonton, Alberta, Canada; The Noujaim Institute for Pharmaceutical Oncology Research, Faculty of Pharmacy and Pharmaceutical Sciences, University of Alberta, Edmonton, Alberta, Canada; Department of Oncologic Imaging, Cross Cancer Institute, Edmonton, Alberta, Canada

**Keywords:** animal tumour model, magnetic resonance imaging, transitional cell carcinoma

## Abstract

An animal tumour model that mimics the human counterpart is essential for preclinical evaluation of new treatment modalities. The objective of this study was to develop and characterize such a model. To accomplish this, the established AY-27 rat bladder transitional cell carcinoma (TCC) cell line was transplanted orthotopically into Fischer CDF344 female rats. AY-27 TCC cells were grown in monolayer cell culture and instilled intravesically as single cell suspensions into bladders that had been conditioned with mild acid washing. Tumour growth was assessed weekly by subjecting the rats to magnetic resonance imaging (MRI). At intervals following implantation and MRI tumour detection, the animals were sacrificed for necropsy, histological examination and immunocytochemical studies. Flow cytometry was also performed for detection of Fas or Fas-ligand expression on AY-27 cells. The overall tumour establishment was 95% (97/102 rats) at 12–50 days, while in a subgroup of animals sacrificed at 16 days, 80 out of 82 animals (97%) developed TCC, the majority of which was superficial. Tumour stage was assessed by gross pathology and light microscopy. Histological examination of the tumour specimens confirmed the presence of grade II–III TCC. Immunocytochemistry confirmed that the tumour model maintained the features of TCC. The changes seen on MRI correlated well with the extent of tumour invasion identified histologically. Patchy carcinoma in situ could be detected histologically 12–13 days post-inoculation, and progressed to papillary tumour or invasive disease thereafter. Neither Fas nor Fas-ligand was expressed on AY-27 cells. The orthotopic AY-27 TCC model is highly reproducible and is ideal for preclinical studies on experimental intravesical therapies. © 1999 Cancer Research Campaign


*British Journal of Cancer* (1999) **81**(4), 638–646
© 1999 Cancer Research Campaign
Article no. bjoc.1998.0741

### Characterization of a novel transplantable orthotopic rat bladder transitional cell tumour model

**Z Xiao[1], TJ McCallum[1], KM Brown[1], GG Miller[2], SB Halls[3], I Parney[1] and RB Moore[1]**

[1]Department of Experimental Surgery and Division of Urology, University of Alberta and Department of Surgery, Cross Cancer Institute, Edmonton, Alberta, Canada; [2]The Noujaim Institute for Pharmaceutical Oncology Research, Faculty of Pharmacy and Pharmaceutical Sciences, University of Alberta, Edmonton, Alberta, Canada; [3]Department of Oncologic Imaging, Cross Cancer Institute, Edmonton, Alberta, Canada

**Summary** An animal tumour model that mimics the human counterpart is essential for preclinical evaluation of new treatment modalities. The objective of this study was to develop and characterize such a model. To accomplish this, the established AY-27 rat bladder transitional cell carcinoma (TCC) cell line was transplanted orthotopically into Fischer CDF344 female rats. AY-27 TCC cells were grown in monolayer cell culture and instilled intravesically as single cell suspensions into bladders that had been conditioned with mild acid washing. Tumour growth was assessed weekly by subjecting the rats to magnetic resonance imaging (MRI). At intervals following implantation and MRI tumour detection, the animals were sacrificed for necropsy, histological examination and immunocytochemical studies. Flow cytometry was also performed for detection of Fas or Fas-ligand expression on AY-27 cells. The overall tumour establishment was 95% (97/102 rats) at 12–50 days, while in a subgroup of animals sacrificed at 16 days, 80 out of 82 animals (97%) developed TCC, the majority of which was superficial. Tumour stage was assessed by gross pathology and light microscopy. Histological examination of the tumour specimens confirmed the presence of grade II–III TCC. Immunocytochemistry confirmed that the tumour model maintained the features of TCC. The changes seen on MRI correlated well with the extent of tumour invasion identified histologically. Patchy carcinoma in situ could be detected histologically 12–13 days post-inoculation, and progressed to papillary tumour or invasive disease thereafter. Neither Fas nor Fas-ligand was expressed on AY-27 cells. The orthotopic AY-27 TCC model is highly reproducible and is ideal for preclinical studies on experimental intravesical therapies. © 1999 Cancer Research Campaign

**Keywords:** animal tumour model; magnetic resonance imaging; transitional cell carcinoma

Bladder cancer is the second most frequent urological malignancy in North America (Parker et al, 1996), and the most common urological cancer in Southeast Asia. More than 90% of patients diagnosed with bladder cancer have transitional cell carcinoma (TCC) (Lamm et al, 1996). Patients with superficial tumours (carcinoma in situ [CIS], Ta, T1) account for 70–85% of all newly diagnosed cases (Whitmore, 1988; Lamm, 1992). The first choice of treatment for stages Ta and T1 disease is transurethral resection (TUR). A major problem in the treatment of superficial TCC of the bladder is the high incidence of tumour recurrence following TUR. In addition to the multifocal origin of these tumours, a further explanation for this high rate of recurrence might be the implantation of tumour cells at the time of resection. In follow-up, patients need periodic cystoscopic examinations and adjuvant intravesical (i.b.) therapies to prevent or postpone tumour recurrence (Grossman et al, 1996). Furthermore, the diagnosis and effective treatment of bladder CIS remain dilemmas.

A suitable bladder tumour model that resembles human disease both histologically and in behaviour is essential for evaluating new therapeutic agents and modalities. The ideal animal bladder tumour model should include the following characteristics:

1. The tumour should grow intravesically (orthotopically), such that the tumour can be directly exposed to i.b. antitumour drugs in its natural environment.
2. The tumour should be of pure TCC origin, with different stages of disease progression (CIS, papillary and invasive diseases) and, as for the human disease, the majority of the tumours should be superficial, but not progressive.
3. The animal host should be immunocompetent and reasonably large, so it can be treated by various antitumour modalities such as immunotherapy with bacillus Calmette–Guérin (BCG), chemotherapy, and whole bladder photodynamic therapy (PDT).
4. The tumour should be technically easy to develop within a reasonable time period, and highly reproducible with respect to its natural history.

Rodent primary bladder tumours have been induced by *N*-[4-(5-nitro-2-furyl)-2-thiazolyl]formamide (FANFT) and other carcinogens for 30 years (Erturk et al, 1967, 1969, 1970; Soloway, 1977; Arai et al, 1979; Ibrahiem et al, 1983; Ohtani et al, 1986; Samma et al, 1990). The induction of primary bladder tumours required 8–11 months, with both TCC and squamous carcinoma induced by carcinogen ingestion (Erturk et al, 1967, 1969, 1970; Herman et al, 1985; Ohtani et al, 1986; Oyasu, 1995; Stocker et al, 1997). A transplantable animal model appears more feasible as an experimental tool for testing new antineoplastic agents. Soloway (1977) showed that bladder tumour cells (MBT-2) could be implanted on the murine bladder mucosa by i.b. tumour inoculation, if the

*Received 11 August 1998*
*Revised 30 November 1998*
*Accepted 7 December 1998*

*Correspondence to:* RB Moore, Department of Surgery, University of Alberta, 2D2.17 Walter Mackenzie Health Sciences Centre, Edmonton, T6G 2R7, Alberta, Canada bladder mucosa was pretreated with *N*-methyl-*N*-nitrosourea (MNU). Ibrahiem and colleagues (1983) injected rat tumour cells into rat bladder muscularis to develop an invasive bladder tumour model, because of tumour cell growth failure when inoculated on the bladder mucosa using Soloway's procedure. Chin et al (1991) established an orthotopic mouse bladder tumour model by implanting MBT-2 tumour cells on bladder mucosa, which was altered by mild acid pretreatment. Compared with Soloway's procedure, the one used by Chin et al was more convenient. Carcinogen could be avoided and higher tumour takes (75–80%) were achieved (Chin et al, 1991). The advantages and limitations of previous animal bladder tumour models were recently reviewed by Oyasu (1995). In mice, most of the deeply invasive bladder tumours were squamous cell carcinomas, as opposed to TCC. The carcinomas observed in mice tended to invade into the perivesical organs by direct extension, and seldom showed metastasis to regional lymph nodes or lung as observed in humans. The rat models, however, were more closely parallel to human bladder carcinomas than mouse models in regards to configuration and progression (Oyasu, 1995). Therefore we adapted the procedure described by Chin et al (1991) by instilling AY-27 TCC cells into syngeneic rat bladders, and monitoring for tumour growth with magnetic resonance imaging (MRI) at weekly intervals. This resulted in efficient establishment of various stages of TCC within a relatively short time period. This orthotopic rat bladder TCC model resembles the human situation resulting from 'seeding' of viable tumour cells. Its characteristics and application potentials are discussed.

### MATERIALS AND METHODS

### Tumour cells and culture conditions

Fischer F344 rats were used to establish the orthotopic bladder tumour model and to propagate the tumour heterotopically (flank or subcutaneous). The rat bladder TCC cell line AY-27 was established as a primary bladder tumour in Fischer F344 rats by feeding FANFT, and was generously provided by Dr Steve Selman at the Medical College of Ohio, Toledo. The cells were initiated from frozen stock and cultured in vitro as monolayers at 37°C in a humidified atmosphere of 5% carbon dioxide in air. RPMI-1640 medium (Gibco-BRL) supplemented with 10% fetal bovine serum, penicillin–streptomycin and 2.0 g $l^{-1}$ sodium bicarbonate, was used throughout. Cells were passaged when nearly confluent by standard, limited trypsinization procedures.

### Preparation of cells

Single tumour cell suspensions were prepared by mincing the tumour under sterile conditions and plating in sterile plastic T-25 flasks (Corning, NY, USA). The medium was decanted from the adherent cells and replaced with fresh medium 4 h later, and at daily intervals for 3–4 days. When the plated cells neared confluence, the growth medium was removed and the cells were incubated for 10 min in Hank's balanced salt solution (HBSS) without $Ca^{++}$ and $Mg^{++}$, supplemented with EDTA (ethylenediaminetetraacetic acid, 200 $mgl^{-1}$, Sigma Chemicals) and antibiotic as above. HBSS was replaced by 0.01% trypsin–EDTA in HBSS and incubated for 10 min. The resulting cell suspension was then centrifuged at 75 ***g*** for 5 min, the supernatant discarded and the cells resuspended in 10 ml HBSS with $Ca^{2+}$ and $Mg^{2+}$. Cell number was determined with a Coulter Counter (Coulter Industries, Model ZBI). Cell suspension directly from cell culture was used for both bladder instillation and subcutaneous injection. For orthotopic implantation, $1.5 \times 10^6$ cells in 0.5 ml HBSS were instilled intravesically. For flank implantation, $1.0 \times 10^6$ cells in 100 μl HBSS were injected subcutaneously. To maintain the phenotypic and cytogenetic fidelity, the AY-27 TCC cell line was passaged periodically as subcutaneous tumours in the flanks of Fischer F344 rats. A tumour cell stock was maintained by cryopreservation.

### Animals

Fischer F344 rats were obtained from Charles River, Canada, and subsequently bred locally in our vivarium. Only female rats (160–200 g) were recipients for the orthotopic bladder inoculation; male Fischer F344 rats were utilized for the flank implantation. All animal procedures were performed in compliance with the Canadian Council on Animal Care Guidelines. Sterile technique was used for tumour cell implantation.

### Tumour implantation

Intravesical instillation of the TCC cell suspension followed that described by Chin et al (1991). Briefly, the rats were anaesthetized with intraperitoneal (i.p.) injections of ketamine (50 mg $kg^{-1}$ body weight, MTC Pharmaceuticals, Cambridge, Ontario, Canada) and xylazine (7.5 mg $kg^{-1}$ body weight, Miles Canada Inc. Etobicoke, Ontario, Canada). Body temperature was maintained with a homeothermic blanket. The rat bladder was catheterized via the urethra with an 18-gauge plastic intravenous (i.v.) cannula (Becton Dickinson, UT, USA). The 18-gauge cannula was optimal for administration of the various agents, providing a snug fit, with no leakage around the catheter. To facilitate tumour seeding, the bladder mucosa was conditioned with an acid rinse. The conditioning consisted of i.b. administration of 0.4 ml of 0.1 N hydrochloric acid (HCl) for 15 s and neutralized with 0.4 ml of 0.1 N potassium hydroxide (KOH) for 15 s. The bladder was then drained and flushed with sterile phosphate-buffered saline (PBS). Histologically, HCl/KOH instillation elicited focal urothelial denudation with slight submucosal blood vessel dilatation. Immediately after bladder conditioning, the AY-27 cells were instilled and left indwelling for at least 1 h. The rats were turned 90° every 15 min to facilitate whole bladder exposure to the tumour cell suspension. Initially, TCC cell inocula were varied from $1 \times 10^6$ to $3 \times 10^6$ cells to examine dose dependency. Unconditioned bladders were examined for tumour takes as well. After 1 h, the catheter was removed and the rats were allowed to void the suspension spontaneously. After recovery from the anaesthetic, the rats were placed into standard cages and monitored daily for haematuria and general health status. Rats were assessed for tumour growth by serial MRI at 8–14 days post-inoculation, and were sacrificed and necropsied at intervals following MRI detection of tumour growth.

To evaluate the natural course of tumour progression over time, 12 rats inoculated with $1.5 \times 10^6$ AY-27 cells were followed until they developed signs of advanced disease. The rats were monitored daily for general health status, and were sacrificed and necropsied when they had any of the following signs: poor grooming, lethargy, hunching, or palpable abdominal mass. All rats were euthanized prior to onset of significant tumour-related morbidity.

© 1999 Cancer Research Campaign

**Table 1** Orthotopic bladder tumour incidence and stage of the 102 rats that received AY-27 cells and were sacrificed at different times post-implantation

| Harvest time (days) | Number of rats | Histology[a] | | | | | | Bladder stone | Number of rats with tumour (%) |
|---|---|---|---|---|---|---|---|---|---|
| | | No tumour | CIS only | T1/CIS[b] | T2 | T3 | T4 | | |
| 12–13 | 8 | 3 | 3 | 2 | 0 | 0 | 0 | 0 | 5/8 |
| 16–17 | 82 | 2 | 17 | 35 | 23 | 5 | 0 | 7 | 80 (97%) |
| 22–50 | 12 | 0 | 0 | 3 | 3 | 4 | 2 | 4 | 12/12 |
| Total | 102 | 5 | 20 | 40 | 26 | 9 | 2 | 11 | 97 (95%) |

[a]CIS: Tumour cells confined to bladder mucosa, no papillary tumour. T1: papillary tumour invading bladder submucosa, but not beyond. T2: papillary tumour invading one-half of the bladder wall, but not through it. T3: papillary tumour penetrating through the bladder wall, no apparent metastasis. T4: papillary tumour invading adjacent organs or metastasizing to distant organs. [b]Papillary tumours were usually accompanied by CIS. If a rat had more than two categories (CIS, T1, T2, T3, and T4), it was allocated to the highest category.

### MRI protocol

For MRI, the rats were anaesthetized with ketamine and xylazine, their bladders were then catheterized and moderately distended with 0.5 ml normal saline. Two rats were simultaneously positioned supine in an MRI knee coil. Turbo STIR (Short-T1 Inversion Recovery sequence) images (TR = 2647, TE = 20, TI = 165) were acquired using 205 × 256 matrix, with a field of view of 100 mm, and 0.49 mm × 0.39 mm in-plane resolution. A Philips Gyroscan ACS-II 1.5T clinical magnet was used to generate 1.8-mm slices with a 0.4-mm interval between slices. No external marker was required to interpret bladder location, which was initially determined by serial scanning of the rats in the transverse and sagittal planes. Typically, STIR images were acquired in both planes, resulting in well defined images capable of monitoring tumours nominally 2 mm in diameter. Imaging of the rats was carried out weekly until sacrificed.

### Gross and microscopic histology

Necropsy was performed on all rats. The extent of tumour burden at necropsy was evaluated by gross and microscopic examination of the bladder, as well as the organs within the cranial, thoracic and peritoneal cavities. The presence or absence of tumour was noted, along with the number of foci, morphology, size and degree of tumour invasion. On removal, the bladders were reflected open and examined for tumour distribution and tumour size. Tissue from the bladders and other organs was fixed in 10% phosphate-buffered formalin, embedded in paraffin, serially sectioned at 4 µm, and were stained with haematoxylin-eosin (H&E) for histological examination.

### Immunocytochemistry of AY-27 bladder tumour

To confirm that the AY-27 tumour has and retains the TCC phenotype after several in vitro and in vivo passages, a three-step, indirect immunoperoxidase staining of paraffin-embedded bladder tumour sections with two monoclonal antibodies directed against cytokeratins 7 and 13 was conducted (Moll et al, 1988). Cytokeratin 7 has been shown to be expressed in normal human and rat urothelia, as well as in transitional cell carcinomas, but not in squamous cell cancer (Moll et al, 1988; Cilento et al, 1994). Cytokeratin 13 is considered to be a reliable 'group marker' for squamous epithelium from different organs, whereas it is absent in pure TCC without squamous differentiation (Moll et al, 1988). Briefly, the 4-µm-thick tissue sections were deparaffinized and heated (Microwave processor, Model H2500, Energy Beam Sciences, Inc.) to 100°C for 10 min, and then treated with 0.05% trypsin for 15 s before adding the antibody. After labelling with the primary antibody (either anti-cytokeratin 7 or 13, mouse $IgG_1$; Boehringer Mannheim Biochemica, Germany), biotin-conjugated second antibody (murine IgG, Dako, USA), and avidin–peroxidase conjugate (Biogenix, Canada), the sections were incubated with diaminobenzidine (DAB), and counter-stained with haematoxylin. For comparison, histological sections of human transitional cell carcinoma, squamous cell carcinoma, rat normal skin and AY-27 cell monolayer cultures, were stained using identical techniques.

To examine for Fas or Fas-ligand (FasL) expression on AY-27 cells, the cells were grown to approximately 70% confluence in T-75 flasks, washed once in PBS with 0.04% EDTA, and then incubated in PBS with 0.04% EDTA for 30 min at 37°C. The cells were then harvested by gentle pipetting, pelleted by centrifugation, washed once with PBS, and again pelleted. The cells were then resuspended in 600 µl immunofluorescence (IF) buffer (1% fetal bovine serum, 0.02% sodium azide in PBS) and 200-µl aliquots were transferred to 1.5 ml Eppendorf tubes and placed on ice. Each aliquoted sample received 1 µl of either anti-Fas (Pharmigen, USA), anti-FasL (Pharmigen, USA), or isotype-matched control (X931, Dako, USA) antibody and was incubated for 1 h on ice. Subsequently, the cells were pelleted, washed twice in IF buffer, and resuspended in 200-µl IF buffer with 10% normal goat serum (Dako, USA). Samples were incubated at room temperature for 10 min, then returned to ice. Each sample received 1 µl of goat anti-mouse-IgG-FITC (Pharmigen, USA) and was then incubated on ice in the dark for 1 h. The cells were pelleted, washed twice in IF buffer, then resuspended in 500 µl of 1% formaldehyde in PBS. Following the above processing, the samples were analysed immediately using a Becton-Dickinson flow cytometer and CellQuest software.

### RESULTS

### Orthotopic bladder tumour establishment

Single-cell suspensions of 1.5 × $10^6$ AY-27 cells instilled into normal (non-conditioned) bladders did not result in tumour establishment. The establishment of bladder tumours following AY-27 cell instillation into bladders with preconditioned mucosa is summarized in Table 1. A total of 106 rats were inoculated intravesically with AY-27 cells. Of these rats, four died of complications related to the anaesthetic or tumour implantation procedure during initial experiments. The overall tumour establishment was

 © 1999 Cancer Research Campaign

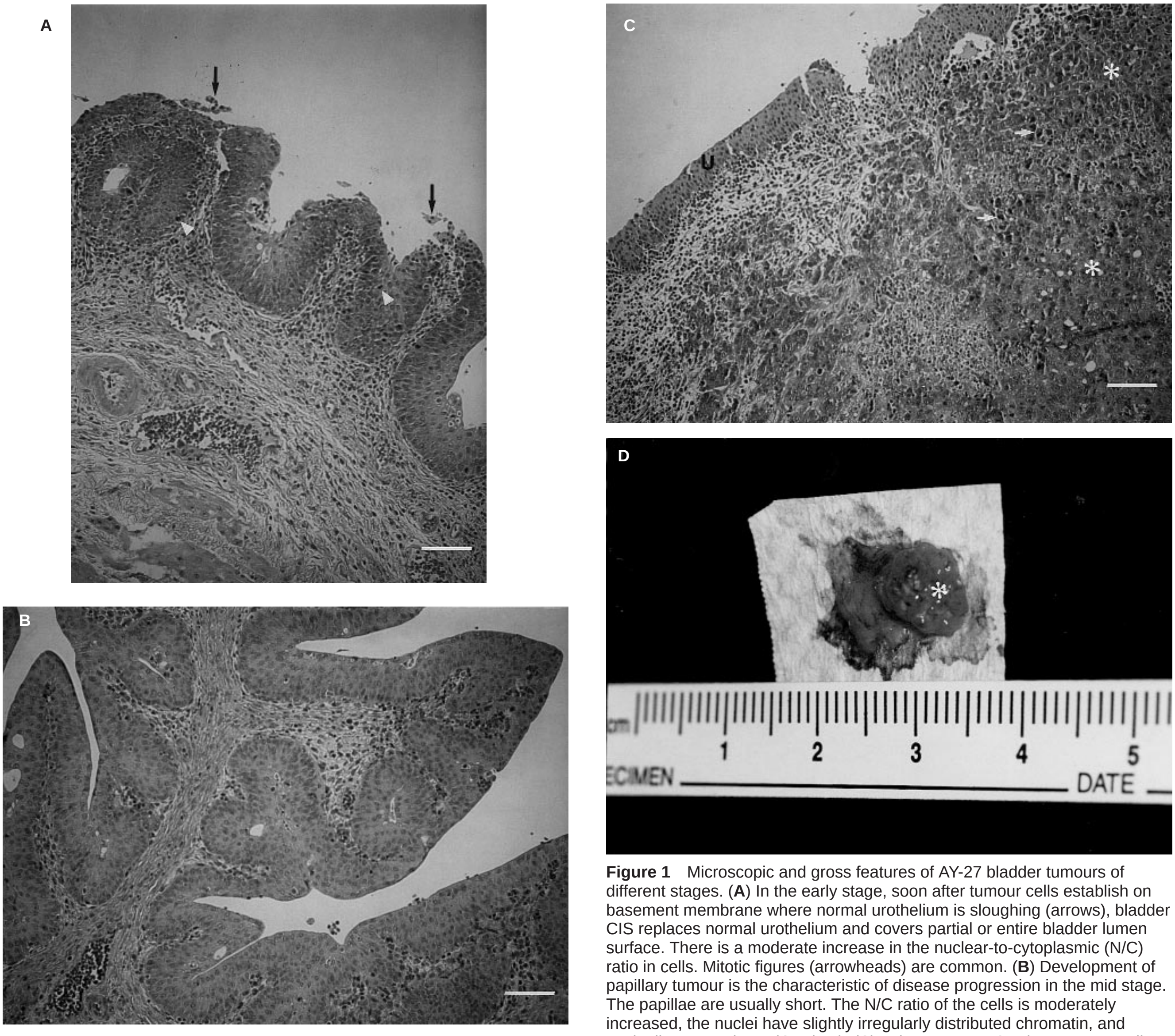

**Figure 1** Microscopic and gross features of AY-27 bladder tumours of different stages. (**A**) In the early stage, soon after tumour cells establish on basement membrane where normal urothelium is sloughing (arrows), bladder CIS replaces normal urothelium and covers partial or entire bladder lumen surface. There is a moderate increase in the nuclear-to-cytoplasmic (N/C) ratio in cells. Mitotic figures (arrowheads) are common. (**B**) Development of papillary tumour is the characteristic of disease progression in the mid stage. The papillae are usually short. The N/C ratio of the cells is moderately increased, the nuclei have slightly irregularly distributed chromatin, and nucleoli are prominent (Grade II). (**C**) In later stages, Grade III tumour cells (*) invade to submucosa and bladder detrusor muscle. There is marked increase in the N/C ratio in cells which form solid sheets. There is markedly increased variation in nuclear size and shape, and mitotic figures (arrows) and nucleoli are frequent and prominent. 'U' denotes normal urothelium. (**D**) Gross view of bladder papillary tumour at mid stage. A solitary tumour (*) locates at bladder posterior-left wall with a cauliflower-like configuration. (**A–C**) H&E, Bar = 100 μm

95% (97/102). Of the 102 remaining rats, eight were sacrificed 12–13 days after tumour cell instillation, three of the eight had no detectable tumour. Carcinoma in situ and/or T1 bladder tumours were detected in the remaining five, and none had developed invasive tumour. Rats inoculated with $1.0 \times 10^6$ AY-27 cells were included in this initial group. Another 82 rats were euthanized on day 16–17 post-implantation following MRI detection of tumour. Only two were tumour-free, 80 rats (97%) developed various stages of bladder tumours: 65% (52/80) with superficial tumours (CIS, T1); 29% with T2; and five out of 80 with T3 bladder tumours. None of these rats developed distant metastases, although two had hydroureteronephrosis, with evidence of secondary infection to tumour obstruction of the ureters. Twelve rats were followed until they were overtly symptomatic, prior to being necropsied on day 22–50 (average 35 days) to assess the natural history of disease progression. All these rats developed bladder tumours, 66% (9/12) of which were invasive T2 to T4 bladder tumours. Hydroureteronephrosis due to tumour obstruction was found in four rats. In two of these four rats, tumour cell metastasis to the common iliac artery lymph nodes was detected. Tumour seeding of the peritoneum was detected in only one rat.

### Gross and microscopic characteristics

Microscopic and gross characteristics of the bladder tumour are presented in Figure 1A–D. The malignant cellular features of this bladder tumour included high mitotic activity, high nuclear-to-cytoplasmic ratio, and marked nuclear pleomorphism. These cellular characteristics are identical to those observed for the AY-27 cells in monolayer culture. Different in vitro passage numbers yielded similar tumour establishment, but with a slight increase in tumour aggressiveness occurring with later passages. The in vitro

© 1999 Cancer Research Campaign

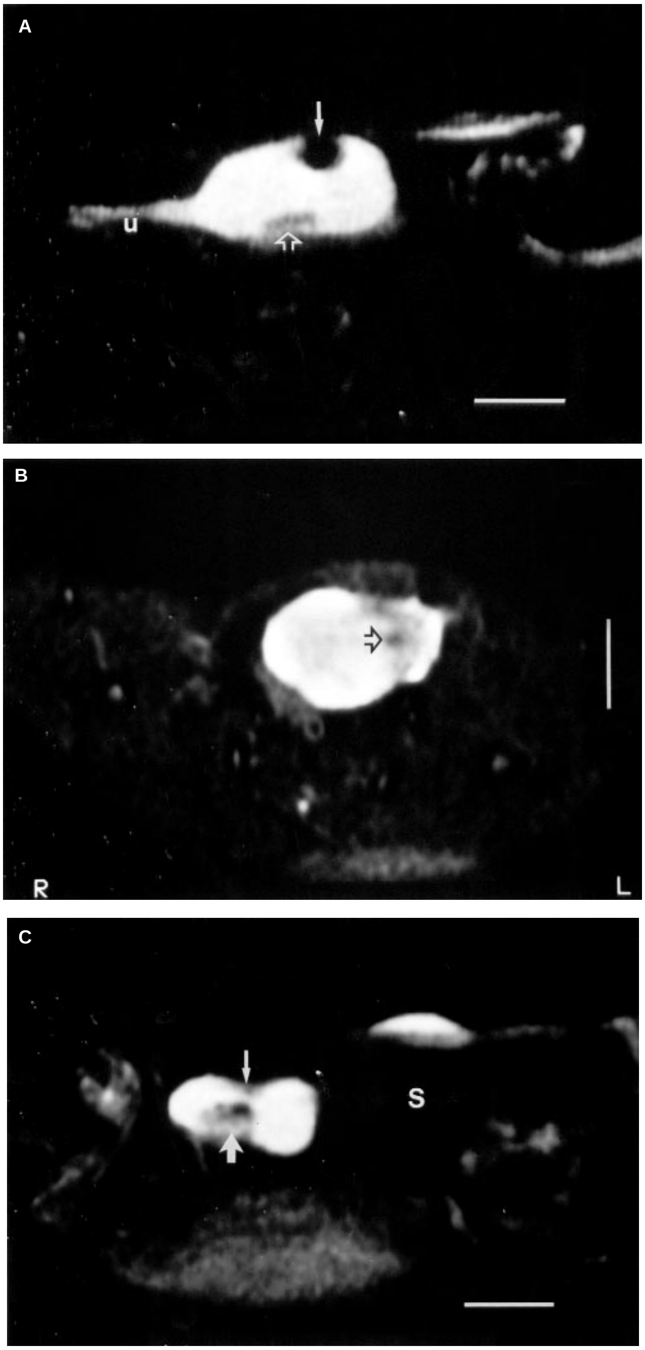

**Figure 2** MR images of rat bladders at different post-inoculation days. (**A**) A thin 'filling defect' lesion (open arrow) lining the posterior bladder wall 13 days following tumour cell instillation. 'u' represents the urethra. There is an air bubble (arrow) locating at the bladder dome. (**B**) A transverse view showing a 'filling defect' lesion (arrow) arising from the posterior left bladder wall, protruding into the lumen 16 days post-implantation. The corresponding tumour is shown in Figure 1D. (**C**) A sagittal view showing the bladder is partially filled with tumour (large arrow) that disrupts the bladder outline (small arrow) with retraction due to tumour invasion at 28 days post-implantation. 'S' denotes the stomach. (**A–C**) Bar = 10 mm doubling time of exponentially growing AY-27 cells was approximately 24 h.

The microscopic and gross features observed after i.b. AY-27 cell inoculations followed a pattern that may be divided arbitrarily into three stages: (1) early tumour establishment (post-inoculation days 1–13); (2) mid stage of i.b. progression (days 14–21); and (3) advanced i.b. progression and extravesical spread (days 22–50). Soon after instillation into the bladder, tumour cells implanted on the basement membrane where the urothelium was denuded. Tumour cells grew and extended along the basement membrane, and eventually replaced the surrounding normal urothelium (Figure 1A). At this early stage, the lesions appeared flat and intra-epithelial, and were defined as bladder CIS.

During the mid stage, these intra-epithelial lesions developed into papillary tumour, or invasive disease, probably depending upon cellular differentiation and host resistance, since in papillary tumour moderately differentiated cells were predominant (Figure 1B), while in invasive tumour, more poorly differentiated cells were prominent (Figure 1C). In some cases, lymphocytic and mononuclear cell infiltration around tumour could be seen. The papillary tumour configuration appeared similar to that of the human counterpart. The tumour papillae, however, were shorter and less branched (Figure 1B), such that the tumour assumed a broad-based cauliflower-like appearance (Figure 1D). In certain cases, diffuse, fine granularity of tumour covering the entire bladder lumen was seen. Twenty-nine per cent of tumour invaded the submucosa connective tissue and/or superficial detrusor muscle. Only in a few cases (5/80) did the tumours invade through the detrusor muscle. Two rats with gross hydroureteronephrosis were found to have evidence of infection secondary to tumour obstruction of the distal ureters.

At the advanced stage, tumour cells penetrated the bladder wall and spread to lymph nodes. The advanced stage was frequently associated with hydroureteronephrosis which also appeared to be caused by direct tumour invasion. Infection following tumour obstruction is the most common life-threatening complication observed in this model. Tumour was detected in the liver in one case, with concurrent massive peritoneal seeding and spread of other visceral organs. In all the rats studied, no evidence of tumour spread was found in the lung, brain, heart and pericardial cavity.

### MRI

Magnetic resonance imaging demonstrated no evidence of tumour until at least 10 days post-implantation. With further experience, we found it only necessary to begin MRI at post-implant day 13. At this time interval, MRI reliably revealed a thin 'filling defect' lesion lining the bladder wall (Figure 2A). By post-implant day 16, part of the bladder lumen was occupied by the 'filling defect' lesion (Figure 2B). Necropsy examination revealed a solitary papillary tumour located on the posterior-left bladder wall (Figure 1D). If invasive tumour developed, MRI showed the bladder outline was disrupted and retracted by the invading tumour (Figure 2C). The changes seen on MRI correlated well with the extent of tumour invasion identified histologically.

### Immunocytochemistry

Both human and rat urothelia were stained strongly positive with anti-cytokeratin 7, while only basal and intermediate cells stained weakly positive with anti-cytokeratin 13. Human TCC cells were strongly positive with anti-cytokeratin 7 staining, but negative with anti-cytokeratin 13 staining. The AY-27 tumour cells from both in vivo bladder tumour and in vitro cell cultures were similarly positive with anti-cytokeratin 7 staining, but negative with anti-cytokeratin 13 (Figures 1C and 3A–B). The AY-27 bladder

*© 1999 Cancer Research Campaign*

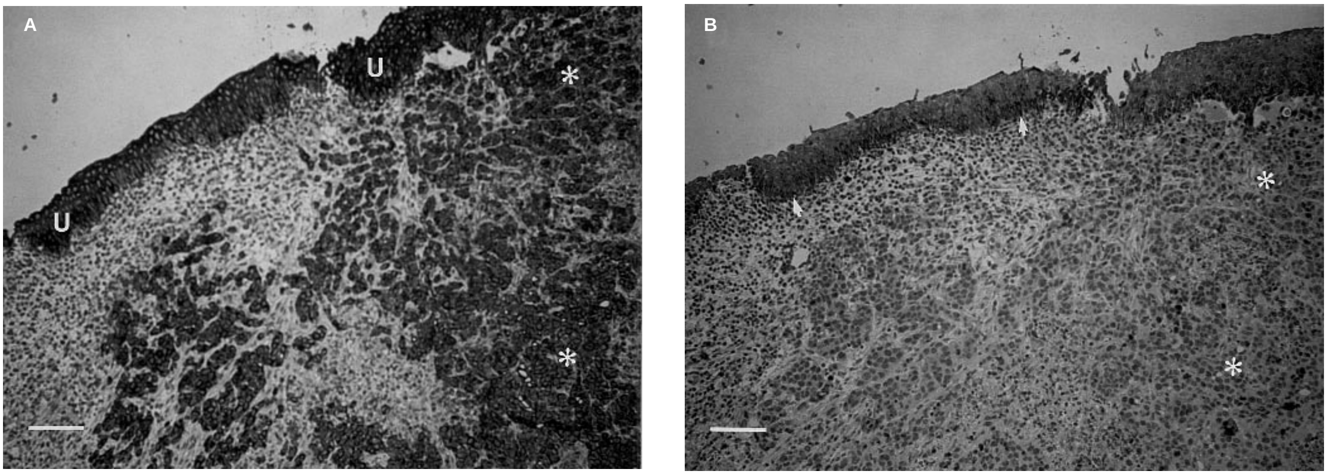

**Figure 3** Immunoperoxidase staining of formalin-fixed, paraffin-embedded rat bladder sections adjacent to the tissue section shown in Figure 1C. (**A**) Staining with anticytokeratin 7 shows that both normal urothelium (U) and tumour cells (*) are positive (dark brown). (**B**) Staining with anti-cytokeratin 13 shows that only basal and intermediate cells (arrows) of normal urothelium are weakly positive (light brown), while tumour cells (*) are negative. Immunochemical staining was performed as described in the Materials and Methods. Bar = 100 μm

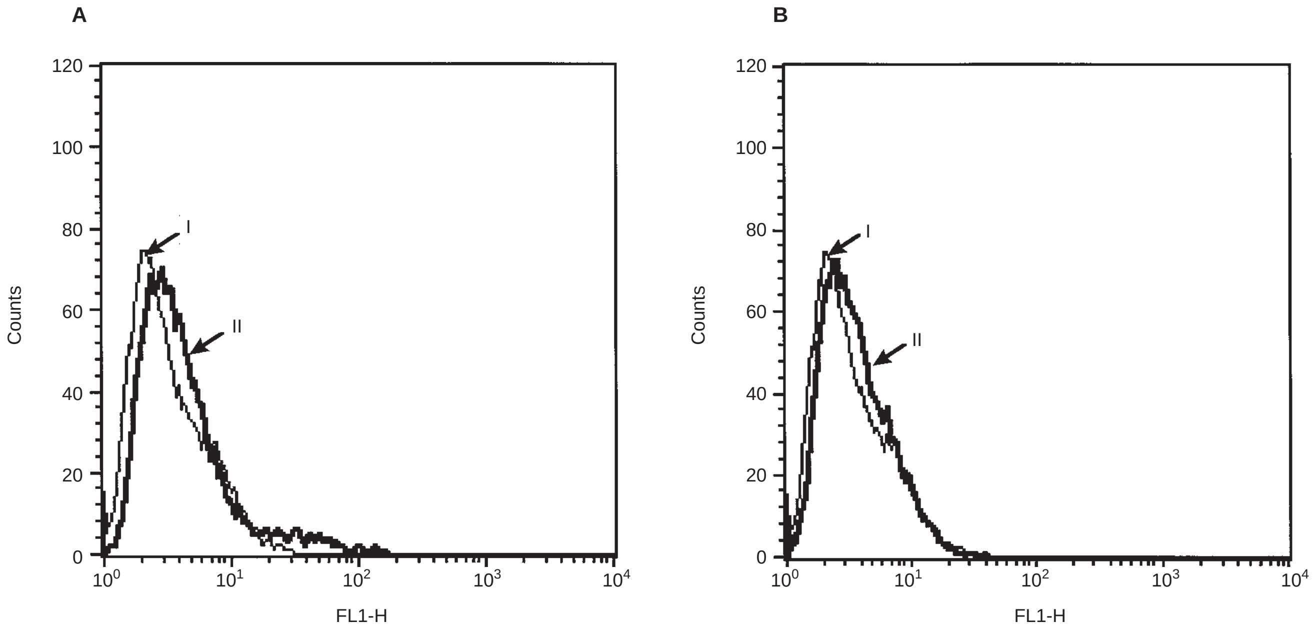

**Figure 4** Fas and FasL expression on AY-27 TCC cells. Cells were incubated first with either anti-Fas (**A**), anti-FasL (**B**), or isotype-matched mouse IgG (control), then with goat anti-mouse-Ig-FITC. Samples were read on the FL 1 parameter using a flow cytometer (see Materials and Methods for details). The fluorescence distribution profiles are not significantly different among control (I) and treated samples (II)

tumour cells used in this model, therefore, maintain urothelial features and demonstrate no evidence of squamous differentiation, both histologically and immunochemically.

Immunofluorescent flow cytometry provided a rapid assay for expression of Fas or FasL. Neither Fas nor FasL is expressed on AY-27 TCC cells, as the fluorescence distribution profiles are not significantly different among control and treated samples (Figure 4).

### Other findings

Eleven rats out of 102 developed small bladder stones or calcific encrustation of the tumour surface. Although the reason for calculus formation was not thoroughly investigated, it was evident that stone formation correlated directly with the presence of tumour necrosis, that occurred with heavy tumour burden. Thus, necrotic tumour appeared to be providing a nidus for stone formation from the lithogenic urine.

Approximately 2 weeks following tumour implantation, some rats failed to gain body weight. These animals maintained their initial body weight, but did not grow as expected, presumably due to tumour growth. In the few animals with advanced stage T4 disease, the tumour mass could be detected by palpation of the lower abdomen.

### Flank tumour growth

Tumour establishment following subcutaneous (s.c.) injection of cell suspension (ten rats), or implantation of 2 $mm^3$ tumour chunks (30 rats) was 100%. Over a period of 4 weeks, the tumours

© 1999 Cancer Research Campaign progressively grew to approximately 15 mm in diameter (about 1700 $mm^3$). The tumour-volume-doubling time was approximately 4 days. Upon dissection, the tumours demonstrated a smooth thin capsule, with a well-vascularized surface and a zone of central necrosis. The tumour architecture was solid, monotonous sheets of TCC cells without papillary growth. No evidence of distant metastasis was found; however, there was evidence of local tumour invasion into the underlying striated muscle in two rats.

### DISCUSSION

Our primary objective was to develop a bladder tumour model for whole bladder PDT and other i.b. therapies. This requires an orthotopic, superficial and purely TCC model with a reasonable bladder volume and urethral caliber to introduce the laser fibre or other therapeutic agents into the bladder. Spontaneously arising rodent bladder tumours are rare (van Moorselaar et al, 1993). A suitable rat model may meet these criteria, since carcinogen induced primary bladder tumours closely parallel the human disease in both morphology and tumour biology (Oyasu, 1995). However, several limitations of previously described models exist. For example, induction of primary bladder tumours requires several months, with both TCC and squamous carcinoma being induced, and tissues other than urothelium also being transformed (Erturk et al, 1967, 1969, 1970; Herman et al, 1985; Oyasu, 1995; Stocker et al, 1997). The subcutaneous tumour model is unsuited to i.b. anti-tumour therapies, not only due to its heterotopic growth, but also due to its dissimilarities in biological behaviour to the clinical disease, as observed in this study. Therefore, ideally, a transplantable orthotopic rat bladder tumour model appears more relevant as an experimental tool for testing new antineoplastic agents provided it is easy to establish and highly reproducible. Soloway (1977) showed that by traumatization of the bladder mucosa by MNU 48 h prior to tumour cell inoculation, the bladder tumour cells (MBT-2) could be implanted intravesically in mouse bladder mucosa. He reported about 60% tumour takes. Ibrahiem and colleagues (1983) injected rat bladder tumour cells into the bladder muscle to develop an invasive bladder tumour model after failure to implant the tumour cells on the bladder mucosa using Soloway's technique. Their model did not closely mimic the human counterpart because the tumour was actually invasive and covered with normal bladder mucosa (Ibrahiem et al, 1983; Harney et al, 1991; Iinuma et al, 1995). Chin and colleagues (1991) reported that tumour cells could be efficiently inoculated in mouse bladder mucosa that was pretreated with mild HCl/KOH. They reported about 80% tumour growth. However, the rat model, because of its size, is easier to work with for i.b. therapies and, furthermore, it may parallel the human disease processes better than a mouse model (Ohtani et al, 1986; Oyasu, 1995). Detailed characteristics of a transplantable orthotopic rat bladder tumour model, to the best of our knowledge, have not been described.

Using the AY-27 cell line and a modification of the techniques previously described by Chin et al (1991) for the MBT-2 model, we have developed and characterized a highly reproducible, transplantable, purely TCC rat orthotopic bladder tumour model. The procedures are not technically complicated, are well tolerated by the animals and result in minimal morbidity associated with occasional mild haematuria subsequent to tumour cell instillation. The fact that tumour cells used for i.b. instillation originated from cell cultures rather than directly from solid tumour fragments, might contribute to the high tumour establishment observed. Tumour cells prepared from tumour fragments may also contain stromal cells, lymphocytes, etc., and their viability might be compromised due to mechanical or enzymatic treatment. Initially, tumour cell suspensions prepared directly from tumour fragments were used for both s.c. injection and i.b. instillation. The resulting tumour grew slower with lower tumour establishment (data not shown). Single-cell suspensions of AY-27 cells instilled in normal (unconditioned) bladder did not elicit tumour establishment. Prior to 13 days post-inoculation, all tumours detected are superficial (T1, CIS). The relatively lower tumour establishment in this group may be explained either by: (1) small early CIS-like lesions escaping detection on histological examination; or (2) this group including three rats inoculated with a lower dose ($1.0 \times 10^6$) of AY-27 cells. In a group of animals sacrificed on days 16–17 post-inoculation, the tumour establishment is 97% (80/82), with 65% being superficial (T1, CIS), 29% superficially invasive (T2), and 6% deeply invasive (T3) disease. Thus 14–16 days post-inoculation seems to be the most suitable time for i.b. therapies such as chemotherapy, immunotherapy or whole bladder PDT. With time, there is progression of the disease process with invasive tumours detected in 66% of the animals followed 21–50 days post-tumour inoculation.

Recently, we also have established an orthotopic nude rat model xerografted with human bladder TCC cells (MGH-U3) using similar procedures described in this study (data not shown). While this tumour model may better represent the cellular biology of human disease, greater time, cost and labour are necessary to maintain the nude rat model than the syngeneic model. As well, the nude model is not appropriate for active immunotherapy like BCG instillation, which currently is the most effective clinical therapy for bladder CIS.

In clinical practice, human bladder carcinoma is commonly classified into three types: CIS, superficial papillary and invasive carcinomas. The superficial papillary tumours frequently recur after TUR, but the prognosis is good. The invasive tumour has a very poor prognosis with the median survival being approximately 1 year despite the use of aggressive regimens (Roth, 1996). Whether the carcinogenesis of these two entities is different has yet to be determined. Studies on animals using bladder carcinogens suggest that both papillary and invasive tumours may be derived from CIS depending on cellular differentiation (Ohtani et al, 1986; Oyasu et al, 1987; Oyasu, 1995). In addition, Orozco et al (1994) analysed 102 patients with primary or secondary bladder CIS, and suggested that host resistance to local progression determined the outcome of CIS, since bladder CIS is cytologically identical to high-grade papillary and invasive TCC. Droller et al (1982) also found that tumour development was accompanied by decreased lymphocyte cytotoxicity against bladder tumour in rats. Our observations in the transplantable tumour model also support these findings. Bladder CIS may develop to papillary or invasive tumours depending on both cellular differentiation and host resistance to disease progression, since the cytology of papillary tumours is similar to that of CIS. The latter is usually thought to be the preliminary stage of invasive diseases. The course of tumour progression observed in our model mimics the clinical situation resulting from viable tumour cell implantation at the time of TUR or prior. These findings may also suggest that the AY-27 cells are heterogeneous.

In addition to cellular differentiation and host resistance, recent evidence shows that cell-to-cell and cell-to-substrate interactions also play an important role in tumour progression. Inhibiting

© 1999 Cancer Research Campaign metastasis by interfering with cancer cell adhesion may be a future therapeutic option, since tumour cells establish bonds with other cells and extracellular matrix by cell adhesion molecules (Ruoslahti, 1996). Cell adhesion molecules consist of multimolecular protein complexes of transmembrane adhesion receptors anchoring intracellular cytoskeletal structural proteins and signal transduction molecules (Yamada et al, 1997). Our model could potentially be utilized to conduct studies of using biological agents to prevent tumour cell seeding by interfering cell adhesion or inducing apoptosis. FasL, a cell surface molecule belonging to the tumour necrosis factor family, binds to its receptor Fas (a type I membrane protein), thus inducing apoptosis of Fas-bearing cells (Nagata et al, 1995). It has been hypothesized that i.b. administration of FasL could be employed as a treatment option for bladder TCC. In the present study, the expression of Fas and FasL on AY-27 cells has been investigated to explore whether the model is suitable for therapy with Fas or FasL. Preliminary evidence suggests that neither marker is expressed in the absence of some form of induction.

Cytokeratins are part of the intermediate filament system of the cytoskeleton (Steinert et al, 1988). The synthesis of cytokeratins is usually maintained during malignant transformation, and this feature serves as one of the hallmarks of epithelium-derived tumours (Tseng et al, 1982), including tumours of the urinary tract (Moll et al, 1982, 1988; Letocha et al, 1993; Cilento et al, 1994). Cytokeratin 7 has been considered a urothelial marker (Cilento et al, 1994), while cytokeratin 13 is a squamous cell marker with trace amounts of component 13 expressed in basal and intermediate cells of human urothelium and low-grade TCC (Moll et al, 1988). Immunocytochemical studies show that the AY-27 rat bladder tumours retain TCC features, without squamous cell differentiation. The latter, if present, usually associates with high-grade (G2–G3), invasive TCC (Oyasu, 1995). Since squamous carcinoma may respond differently to anticancer therapies than TCC carcinoma, this purely TCC tumour model is ideal for preclinical evaluation of anti-tumour therapies for human bladder TCCs, the most common type of cancers arising from the urothelium.

MRI provides a non-invasive imaging modality that is useful to detect early superficial papillary tumour and monitor tumour growth in rodent models. Previously, confirmation of successful tumour implantation in rodents was based on the detection of a palpable suprapubic mass, gross haematuria, weight loss in the animals, or on a transillumination technique by periodic surgical exposure of the bladder (Ibrahiem et al, 1983). In the present study, only two rats with advanced (stage T4) tumours had a palpable suprapubic mass. The average weight loss was insignificant (0.2%). The changes shown on MRI correlated well with the extent of tumour invasion identified histologically. However, once this tumour model had been characterized for growth kinetics, regular monitoring with MRI added little benefit because of the high reproducibility of this model.

In conclusion, the distinct advantageous features of this model system include: (1) purely TCC carcinomas growing orthotopically in the bladder lumen; (2) highly reproducible tumour growth in a relatively short time; (3) resemblance to high-grade human TCC in both morphology and tumour biology; and (4) utility to test varying intravesical therapies such as chemotherapy, active and passive immunotherapies and whole bladder PDT. This model has been used for PDT (Xiao et al, 1998) and BCG immunotherapy of bladder cancer in our laboratory.

### ACKNOWLEDGEMENTS

The authors acknowledge funding support from the Alberta Heritage Foundation for Medical Research, the Edmonton Civic Employees Fund, the Departments of Surgery and Experimental Surgery, University of Alberta, and the Alberta Cancer Board. We thank Irene I Piva, RTR.CMR, and Doris Jeske, RTR.CMR for excellent technical support with MRI imaging, Mr Laith Dabbagh for excellent assistance with immunocytochemical staining, Judith Hugh, MD, for consultation on pathology studies, and the staff of the Cross Cancer Institute Vivarium for expert animal care. The anti-cytokeratin 7 was generously provided by Dr Judith Hugh, Department of Pathology, Cross Cancer Institute, Edmonton. The anti-cytokeratin 13 was a gift from Dr David Rayner at the Department of Pathology, University of Alberta Hospitals, Edmonton, Canada.

### REFERENCES

Arai M, Cohen SM, Jacobs JB and Friedell GH (1979) Effect of dose on urinary bladder carcinogenesis induced in F344 rats by *N*-[4-(5-nitro-2-furyl)-2-thiazolyl]formamide. *J Natl Cancer Inst* **62**: 1013–1015

Chin J, Kadhim S, Garcia B, Kim YS and Karlik S (1991) Magnetic resonance imaging for detecting and treatment monitoring of orthotopic murine bladder tumour implants. *J Urol* **145**: 1297–1301

Cilento BG, Freeman MR, Schneck FX, Retik AB and Atala A (1994) Phenotypic and cytogenetic characterization of human bladder urothelia expanded in vitro. *J Urol* **152**: 665–670

Droller M and Gomolka D (1982) Expression of the cellular immune response during tumor development in an animal model of bladder cancer. *J Urol* **128**: 1385–1389

Erturk E, Price, JM, Morris JE, Cohen S, Letth RS, Von Esch AM and Crovetti AJ (1967) The production of carcinoma of the urinary bladder in rats by feeding *N*-[4-(5-Nitro-2-furyl)-2-thiazolyl]formamide. *Cancer Res* **27**: 1998–2002

Erturk E, Cohen S, Price JM and Bryan GT (1969) Pathogenesis, histology, and transplantability of urinary bladder carcinomas induced in albino rats by oral administration of *N*-[4-(5-nitro-2-furyl)-2-thiazolyl]formamide. *Cancer Res* **29**: 2219–2228

Erturk E, Cohen S and Bryan GT (1970) Urinary bladder carcinogenicity of *N*-[4-(5-nitro-2-furyl)-2-thiazolyl]formamide in female Swiss mice. *Cancer Res* **30**: 1309–1311

Grossman HB (1996) Superficial bladder cancer: decreasing the risk of recurrence. *Oncology* **10**: 1617–1624

Herman C, Vegt PDJ, Debruyne FMJ and Ramaekers FCS (1985) Squamous and transitional elements in rat bladder carcinomas induced by *N*-butyl-*N*-4-hydroxybutyl-nitrosamine (BBN). A study of cytokeratin expression. *Am J Pathol* **120**: 419–426

Ibrahiem EI, Nigam VN, Brailovsky CA, Madarnas P and Elhilali M (1983) Orthotopic implantation of primary *N*-[4-(5-Nitro-2-furyl)-2-thiazolyl]formamide-induced bladder cancer in bladder submucosa: an animal model for bladder cancer study. *Cancer Res* **43**: 617–622

Iinuma S, Bachor R, Flotte T and Hasan T (1995) Biodistribution and phototoxicity of 5-aminolevulinic acid-induced PpIX in an orthotopic rat bladder tumour model. *J Urol* **153**: 802–806

Lamm DL (1992) Superficial bladder cancer. *Urol Clin North Am* **19**: 3

Lamm DL and Torti FM (1996) Bladder cancer, 1996. *CA Cancer J Clin* **46**: 93

Letocha H, Nilsson S, Silen A, Ekblom J, Arnberg H, Wiklund B and Westlin J-E (1993) Immunotargeting with monoclonal cytokeratin 8 antibodies of human urothelial cancer transplanted to nude mice. *Acta Oncol* **32**: 793–800

Moll R, Franke WW, Schiller DL, Geiger B and Krepler R (1982) The catalog of human cytokeratins: patterns of expression in normal epithelia, tumours and cultured cells. *Cell* **31**: 11–24

Moll R, Achtstatter T, Becht E, Balcarova-Stander J and Franke WW (1988) Cytokeratins in normal and malignant transitional epithelium: maintenance of expression of urothelial differentiation features in transitional cell carcinomas and bladder carcinoma cell culture lines. *Am J Pathol* **132**: 123–144

Nagata S and Golstein P (1995) The Fas death factor. *Science* **267**: 1449–1456

Ohtani M, Kakizoe T, Nishio Y, Sato S, Sugimura T, Fukushima S and Niuima T (1986) Sequential changes of mouse bladder epithelium during induction of invasive carcinomas by *N*-butyl-*N*-(4-hydroxybutyl)nitrosamine. *Cancer Res* **46**: 2001–2004

© 1999 Cancer Research Campaign 

Orozco R, Martin AA and Murphy WM (1994) Carcinoma in situ of the urinary bladder. Clues to host involvement in human carcinogenesis. *Cancer* **74**: 115–122

Oyasu R (1995) Epithelial tumors of the lower urinary tract in humans and rodents. *Fd Chem Toxic* **33**: 747–755

Oyasu R, Samma S, Ozono S, Bauer K, Wallemark CB and Homma Y (1987) Induction of high-grade, high-stage carcinomas in the rat urinary bladder. *Cancer* **59**: 451–458

Parker SL, Tong T, Bolden S and Wingo PA (1996) Cancer statistics, 1996. *CA Cancer J Clin* **46**: 1

Roth BJ (1996) Chemotherapy for advanced bladder cancer. *Semin Oncol* **23**: 633–644

Ruoslahti E (1996) How cancer spreads. *Sci Am* (September): 72–77

Samma S, Uchida K, Seidenfeld J and Oyasu R (1990) Effect of α-difluoromethylornithine on the development of deeply invasive urinary bladder carcinomas in mice. *Urol Res* **18**: 277–280

Soloway MS (1977) Intravesical and systemic chemotherapy of murine bladder cancer. *Cancer Res* **37**: 2918–2929

Steinert PM and Roop DR (1988) Molecular and cellular biology of intermediate filaments. *Annu Rev Biochem* **57**: 593–625

Stocker S, Knuchel R, Sroka R, Kriegmair M, Steinbach P and Baumgartner R (1997) Wavelength dependent photodynamic effects on chemically induced rat bladder tumours following intravesical instillation of 5-aminolevulinic acid. *J Urol* **157**: 357–361

Tseng SCG, Jarvinen MJ, Nelson WG, Huang J-W, Woodcock-Mitchell J and Sun T-T (1982) Correlation of specific keratins with different types of epithelial differentiation: monoclonal antibody studies. *Cell* **30**: 361–372

Van Moorselaar RJ, Ichikawa T, Schaafsma HE, Jap PH, Isaacs JT, Van Stratum P, Ramaekers FC, Debruyne FM and Schalken JA (1993) The rat bladder tumor model system RBT resembles phenotypically and cytogenetically human superficial transitional cell carcinoma. *Urol Res* **21**: 413–421

Whirmore WF Jr (1988) Bladder cancer: an overview. *CA Cancer J Clin* **38**: 213

Xiao Z, Miller GG, McCallum TJ, Brown KM, Lown JW, Tulip J and Moore RB (1998) Biodistribution of Photofrin II and 5-aminolevulinic acid-induced protoporphyrin IX in normal rat bladder and bladder tumour models: implications for photodynamic therapy. *Photochem Photobiol* **67**: 573–583

Yamada KM and Benjamin G (1997) Molecular interactions in cell adhesion complexes. *Cur Opin Cell Biol* **9**: 76–85

*© 1999 Cancer Research Campaign*
